# Effects of indoor pollution on acute respiratory infections among under-five children in India: Evidence from a nationally representative population-based study

**DOI:** 10.1371/journal.pone.0237611

**Published:** 2020-08-14

**Authors:** Dinabandhu Mondal, Pintu Paul

**Affiliations:** Centre for the Study of Regional Development, School of Social Sciences, Jawaharlal Nehru University, New Delhi, India; The Ohio State University, UNITED STATES

## Abstract

**Objective:**

Acute respiratory infections (ARI) are the leading causes of neonatal and child mortality. Despite several national efforts to reduce the incidence of mortality among children, India is one of the largest contributors to under-five mortality in the world. In this study, we examined the effects of indoor pollution on ARI among under-five children in India.

**Methods:**

A cross-sectional study was carried using nationally representative data from the 2015–2016 National Family Health Survey (NFHS-4). This study is based on 247,743 living children under the age of five years. Bivariate and multivariate analyses were performed to assess the impact of indoor air pollution on children's ARI.

**Results:**

Almost two-thirds of households (65.2%) used biomass fuels for cooking, 54.9% of households had a separate kitchen, and 47.2% of households had a smoker. About 2.7% of children suffered from ARI in the past two weeks preceding the survey. The use of biomass fuels (OR [odds ratio]: 1.10, 95% CI: 1.01–1.20), households having no separate kitchen (OR: 1.22, 95% CI: 1.14–1.30), and smoking behavior of household members (OR: 1.06, 95% CI: 1.00–1.12) were associated with greater risk of ARI among under-five children even after adjusting for age of child, sex of child, birth order, maternal age, maternal education, caste, religion, wealth quintile, any HH members suffer from tuberculosis (TB), and household crowding. Furthermore, the results revealed that the combined effects of biomass fuels and households without separate kitchen increased the likelihood of children's ARI by 36% (Adjusted OR: 1.35, 95% CI: 1.21–1.51).

**Conclusion:**

The findings of this study suggest policy interventions to reduce the exposure of indoor air pollution, particularly among the impoverished groups. The government should ensure cleaner fuels for cooking, such as LPG and electricity, to minimize the risk of respiratory diseases among children.

## Introduction

Acute respiratory infections (ARI) are the most common causes of childhood morbidity and mortality in the world [1, 2]. Respiratory infections, together with diarrheal diseases, remain significant causes of hospital admission among children in low- and middle-income countries. Although substantial progress has been observed in reducing under-five mortality over the past two decades, pneumonia, which is a severe form of acute lower respiratory infections, appears as a common reason for avoidable neonatal and child mortality [3–5]. As per recent estimates of the World Health Organization (WHO), around 809 thousand deaths of under-five children occurred due to pneumonia, accounting for 15% of all childhood mortality worldwide [6]. Most of these deaths have been reported in sub-Saharan African and South Asian countries [2]. In India, despite interventions for prevention and control of respiratory infections under the Reproductive and Child Health (RCH) program since 1992, pneumonia is the single largest contributor to under-five mortality (17.1%) in the country [7]. In a study of cause-specific mortality, Million Death Study Collaborators (2010) reported that approximately 369 thousand deaths occurred in India because of pneumonia among children of 1–59 months at the rate of 13.5 per 1000 live births [8]. Evidence found that 30–50% outpatient department (OPD) attendance, as well as 20–40% hospital admission may be attributed to respiratory infections [9].

Reducing the preventable deaths of newborns and under-five children is one of the Sustainable Development Goals (SDGs), especially targeting neonatal mortality to at least as low as 12 per 1000 live births and under-five mortality to less than 25 per 1000 live births by 2030 [10]. Achievement of this SDG target could only be possible after averting the deaths of children caused by preventable diseases, particularly pneumonia, which accounts for 15% of all childhood deaths. Despite a disease of such magnitude and severity worldwide, only about one in five caregivers knows about the danger signs of pneumonia, and a little knowledge is available about the detailed epidemiology of respiratory infections [1, 11].

Indoor air pollution is one of the significant risk factors of pneumonia among children. Indoor pollution is caused by cooking and heating with biomass fuels. Biomass fuels, such as woods, straw/shrubs/grass, agricultural crops, animal dung, etc. lie at the lower end of the energy ladder in terms of combustion efficiency and cleanliness. The combustion of these fuels produces a large number of harmful pollutants, such as carbon monoxide, sulfur and nitrogen oxides, 1,3-butadiene, benzene, polycyclic aromatic hydrocarbons, particulate matter, and other toxic organic compounds [12]. In India, like other developing countries, access to cleaner energy is restricted to a few people as only 5% of households use LPG gas exclusively, and 72% of households still use traditional biomass fuels for cooking [13]. The burning of biomass fuels mostly in simple and unvented household cook-stoves compounded with poor ventilation facility generates high levels of indoor air pollution [14, 15].

The daily combustion of biomass materials and exposure to air pollution often goes beyond the standard limit recommended by the World Health Organization [16]. Women of the household, who used to do most of the cooking job and children, who mostly remain within the house especially with mothers while cooking, are profoundly affected by the smoke and pollutants coming out of cooking [17, 18]. Therefore, exposure to indoor pollution is usually higher among women and children. Unlike the adults, the children of young age with immature nasal structure and lesser immunity become more vulnerable while inhaling the polluted air [19]. Therefore, children easily get affected by various respiratory infections, which even sometimes become the reason for their death. Several studies conducted in Nigeria [20], Kenya [21], Bangladesh [22], Brazil [23], and Ethiopia [24] have found a strong association between indoor pollution from biomass fuels and acute respiratory infections among children. Studies in India [25–27] also found that cooking smoke has a substantial impact on children's respiratory illness. However, the impact of indoor pollution on respiratory infections of children has been poorly understood in India, where most of these previous studies have mainly focused on the combustion of biomass fuels in determining the risk of respiratory illnesses among children, and also the findings are based on much older evidence. Moreover, the accessibility of separate kitchen in the household and environmental tobacco consumption has largely been ignored in childhood respiratory illness research of earlier studies, especially in India. In this backdrop, the present study would provide fresh evidence for policy interventions to reduce the susceptibility of respiratory infections among children caused by household air pollution. Therefore, this study attempts to assess the effect of indoor air pollution on acute respiratory infections of under-five children in India, using a recent nationally representative large-scale survey.

## Materials and methods

### Data source

Data were drawn from the latest demographic and health survey in India, popularly known as National Family Health Survey-4 (NFHS-4), conducted in 2015–16. The NFHS-4 is a nationally representative large-scale sample survey comprising 601,509 households, 699,686 women aged 15–49 years, and 112,122 men aged 15–54 years. The survey was carried out across all the 29 states and seven union territories in the country. The samples were collected using a stratified two-stage design. The aim of this large-scale survey was to provide updated information on key population indicators, health and nutrition status, and a range of health-related issues including maternal and child health, fertility, mortality, morbidity, nutritional status, immunization, family planning methods, etc. up to the district level. Details of sample design, including sampling framework and sample implementation, are provided in the national NFHS-4 report [28].

### Study participants

**Fig 1:**
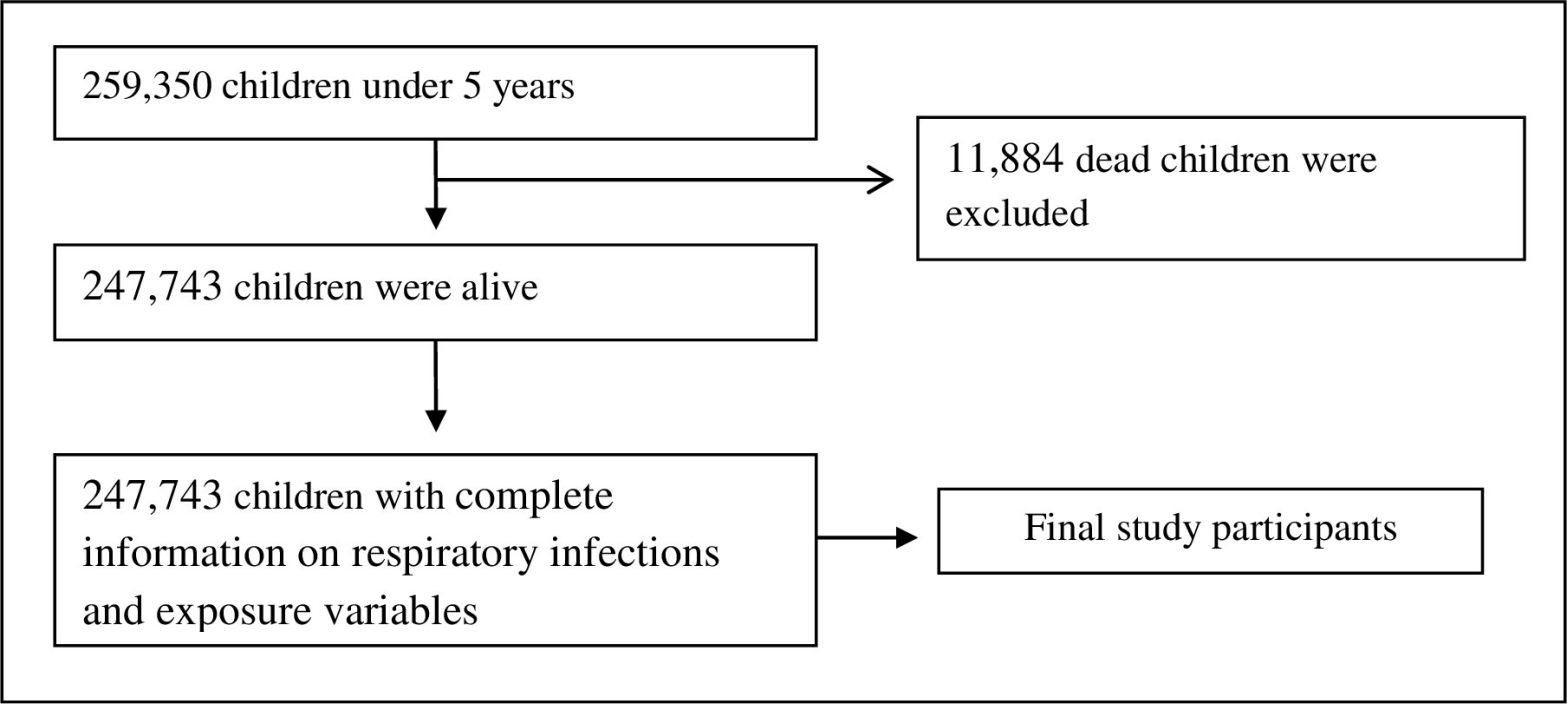
Selection of study participants.

There were 259,627 ever-born children under five years of age in the NFHS-4. Out of these children, 247,743 children were alive, and 11,884 were found dead during the survey. These dead children were excluded as information regarding respiratory infections was not available for them. In this study, 247,743 living children under five years of age, who had complete information on respiratory infections and exposure variables, were selected as study participants (Fig 1). In the respective households of these children, the respondents were asked about the symptoms of acute respiratory infections among children and household information regarding the use of cooking fuel, the presence of separate kitchen and smoking behaviors of household members, etc. A cross-sectional study design was adopted using the information of these children.

### Outcome variable

The outcome variable of this study is acute respiratory infections among children under five years of age. In the NFHS-4, ARI was defined as cough accompanied by short, rapid, or difficult breathing that is chest related. Mothers were asked whether their children had symptoms of ARI within the two weeks prior to the survey. For the analysis in this study, ARI among children was dichotomized into yes (coded as 1) and no (coded as 0). If children suffered from any symptoms of cough or difficulty in breathing in the past two weeks were coded as '1' and children who did not experience any symptoms of ARI as '0'.

### Exposure variables

Indoor air pollution is the exposure variable of this study. Indoor air pollution of the household was indirectly ascertained by the type of fuel used for cooking, facility of kitchen, and smoking behavior of household members. These three variables have been utilized as proxy measure of indoor air pollution. The extent of emission of pollutants from households directly varies with the kind of energy used for cooking, where solid fuels produce more pollution per cooked meal compared to the liquid or gaseous fuels [29]. Therefore, the type of fuel could be treated as a good indicator of indoor pollution. Information regarding cooking fuel was collated from the NFHS-4. In this survey, a question, *"what type of fuel does your household mainly use for cooking*?*"* was asked to the respondents. A detailed list of different types of cooking fuels used in the household was captured as electricity, liquid petroleum gas (LPG) and natural gas, biogas, kerosene, coal and lignite, charcoal, wood, straw/shrubs/grass, agricultural crop, animal dung, and others. We grouped the household usage of fuels into two categories–(1) clean fuels comprising electricity, liquid petroleum gas (LPG), natural gas, and biogas and (2) biomass fuels which include kerosene, coal and lignite, charcoal, wood, straw/shrubs/grass, agricultural crop, animal dung, and others. Households with clean fuels are expected to be less exposed to indoor pollution, while households using biomass fuels are at higher risk of pollution. Information about the kitchen was captured from the survey by asking: *"do you have a separate room which is used as a kitchen*?*"* A variable, separate kitchen was created and dichotomized as households having a separate kitchen (coded as '1') and households without a separate kitchen (coded as '0'). The understanding is that households without a separate kitchen would be more prone to indoor pollution. The survey also provided information regarding the smoking behavior of household members. The frequency of smoking by the members of the household was recorded as daily, weekly, monthly, less often than once a month, and never. Exposure to smoking was indicated as '1' (yes) if any household member smokes inside the house and '0' (no) otherwise.

### Confounding variables

Several socio-demographic variables were included as confounding factors that have a potential impact on the prevalence of respiratory illness. The confounding variables of this study include age of child (0–11, 12–35, 36–59 months), sex of child (male, female), birth order (<3, ≥3), maternal age (15–24, 25–34, 35–49 years), maternal education level (illiterate, primary, secondary, higher), caste (Scheduled Caste [SC]/Scheduled Tribe [ST], Other Backward Classes [OBC], others), religion (Hindu, Muslim, others), wealth quintile (poorest, poorer, middle, richer, and richest), history of household members suffering from tuberculosis (no, yes), and household crowding (≤3 persons per room, >3 persons per room).

It is worthwhile to mention that wealth quintile used as a proxy measure of standard of living. Wealth quintile was calculated using a range of consumer items (e.g., car, bicycle, radio, television, etc.) and housing characteristics (e.g., drinking water, sanitation facilities, housing materials, etc.). A score was derived for each individual using principal component analysis and grouped into five quintiles, each quintile represents 20% of the respondents, ranging from 1 (poorest) to 5 (richest).

### Method of analysis

Descriptive statistics were carried out to understand the characteristics of study participants. Bivariate percentage distribution was estimated to assess the prevalence of ARI among children by the key predictors and confounding variables, and the differences were later tested by Pearson's chi-square statistic. The sample weight was used for the estimation of percentage distribution. A series of binary logistic regression models were employed to examine the association between indoor pollution and ARI among children. In the first model, the crude association was estimated between the type of fuel and ARI of children. A separate kitchen variable was added to the second model. In the third model, the smoking behavior of HH members was progressively included. Finally, socio-demographic characteristics (i.e., age of child, sex of child, birth order, maternal age, maternal education, caste, religion, wealth quintile, history of HH members suffer from TB, and household crowding) were incorporated in the full model to estimate the net effect of indoor pollution on the prevalence of ARI among under-five children. We hypothesized that children living in households using biomass fuels for cooking and without a separate kitchen are more likely to be exposed to indoor pollution, which ultimately results in a higher prevalence of ARI among children. We carried out an additional analysis applying an interaction term between the type of cooking fuel and separate kitchen to test whether exposure to biomass fuels and no separate kitchen modifies the effect of indoor pollution on the occurrence of ARI among children. The regression results were presented in the form of odds ratios (ORs) with 95% confidence intervals (CIs). All the statistical analyses were performed using STATA version 14.0 (StataCorp LP, College Station, TX, USA).

### Ethical statement

This study used secondary data drawn from the National Family Health Survey 2015–16 (NFHS-4). The Ethics Review Board at the International Institute for Population Sciences, Mumbai, India granted this NFHS-4 project ethical approvals before the surveys were conducted, with written informed consent obtained from participants during the surveys. This survey was also reviewed and approved by ICF International Review Board (IRB). The NFHS-4 is an anonymous publicly available dataset with no identifiable information of the survey participants and the datasets are accessible from the Demographic Health Survey (DHS) program at https://dhsprogram.com/data/available-datasets.cfm. Therefore, no separate ethical approval is required for this study.

## Results

### Participant's characteristics

Among 247,743 living under-five children, about 2.7% suffered from ARI in the past two weeks preceding the survey. Almost two-thirds of households (65.2%) used biomass fuels for cooking. Over half of the households (54.9%) had a separate kitchen for cooking. Regarding smoking behavior, almost every second member of the family (47.2%) had smoking habits. In child demographics, 19.3% were infants, 52.1% were males, and 29.1% of children were in the birth order of three or above. In maternal characteristics, over one-third of mothers (34.9%) were in the younger age group (aged 15–24 years). A substantial proportion (29.6%) of mothers had no formal education. The majority of the children belonged to Other Backward Classes (46.1%), affiliated to Hindu religion (78.6%), and were from the poorest (24.9%) and poorer (21.8%) quintiles of household wealth. About 1.6% of children belonged to the households where any family member suffered from TB. Over half of the households (56.8%) lived in crowded houses where more than three persons stayed together per room (Table 1).

**Table 1:** Prevalence of Acute Respiratory Infections (ARI) among under-five children by indoor air pollution and socio-demographic characteristics in India, 2015–16.

Variables	Sample characteristics		ARI prevalence (%)	P-value
	Frequency (n)	Percentage (%)		
***Outcome variable***				
ARI				
No	240,783	97.3		
Yes	6,960	2.7		
***Key predictors***				
Type of cooking fuel				0.003
Clean fuel	164,693	65.2	2.9	
Biomass fuel	69,913	34.8	2.4	
Separate kitchen				<0.001
Yes	90,181	45.1	3.1	
No	112,002	54.9	2.4	
Smoking behavior				<0.001
No	120,623	52.8	2.6	
Yes	127,120	47.2	2.9	
***Confounding variables***				
Age of children (months)				<0.001
0–11	48,295	19.3	3.4	
12–35	98,368	39.9	3.0	
36–59	101,080	40.9	2.2	
Sex of children				<0.001
Male	128,609	52.1	3.0	
Female	119,134	47.9	2.5	
Birth order				0.001
<3	168,600	70.9	2.6	
≥3	79,143	29.1	3.0	
Maternal age (years)				<0.001
15–24	80,714	34.9	3.0	
25–34	142,212	56.7	2.6	
35–49	24,817	8.4	2.7	
Maternal education				0.003
No education	76,212	29.6	2.7	
Primary	35,917	13.9	3.0	
Secondary	112,227	45.8	2.8	
Higher	23,387	10.7	2.4	
Caste				<0.001
SC/ST	96,290	33.4	2.8	
OBC	97,011	46.1	2.7	
Other	43,329	20.5	2.7	
Religion				<0.001
Hindu	178,712	78.6	2.6	
Muslim	39,004	16.6	3.2	
Other	30,027	4.9	2.5	
Wealth quintile				<0.001
Poorest	64,443	24.9	3.1	
Poorer	58,294	21.8	2.9	
Middle	49,588	19.9	2.7	
Richer	41,472	18.4	2.6	
Richest	33,946	15.1	2.3	
Any HH member suffers from TB				0.033
No	243,728	98.4	2.7	
Yes	4,015	1.6	3.3	
Household crowding				<0.001
≤3 persons per room	114,309	43.2	2.7	
>3 persons per room	131,944	56.8	2.8	

P-value: Significance level of Pearson's Chi-square statistic.

### Prevalence of ARI among under-five children by explanatory variables

The ARI prevalence was higher among children living in households using biomass fuels compared to those living in households using cleaner fuels (2.9% vs. 2.4%, *p* <0.03). Children's ARI was lower for households having a separate kitchen facility compared to those who did not have it (2.4% vs. 3.1%, *p* <0.001). A significantly higher proportion of children suffered from ARI, where any family member smoked within house compared to those having no smoking behavior among family members (2.9% vs. 2.6%, *p* <0.001). The occurrence of ARI was higher among infants (3.4%), male children (3%), and birth order of three or above (3%). The prevalence of ARI among children was found to be decreasing with the increasing age of mothers. Women with higher education had a lower proportion of ARI among children compared to those who had a secondary or lower level of education. The occurrence of ARI was higher among children who belonged to SC/ST (2.8%) and Muslims (3.2%). The experience of ARI among children decreased from the bottom to upper wealth quintiles. ARI prevalence was higher among children whose household members were suffering from TB than those households having no TB patient (3.3% vs. 2.7%, *p* <0.033). Furthermore, the incidence of ARI was slightly higher among children who stayed in crowded households that those who had less than three persons living per room (2.8% vs. 2.7%, p<0.001) (Table 1).

### Impact of indoor air pollution on ARI of under-five children

Table 2 presents the binary logistic regression models for assessing the impact of indoor air pollution on children's ARI. Model 1 shows the crude association between the type of cooking fuel and ARI prevalence of under-five children. It is observed that the usage of biomass fuels was associated with an increased likelihood of children's ARI. In model 2, after adjusting for the separate kitchen variable, biomass fuel remained significantly associated with an elevated risk of children's ARI. Similarly, children living in households without a separate kitchen were more likely to suffer from ARI compared to those households having a separate kitchen. In model 3, the smoking behavior variable was progressively included. It is found that the smoking behavior of household members significantly increased the risk of ARI in children. In model 4, socio-demographic characteristics were included as potential controlling factors to assess the net impact of indoor air pollution on children's ARI prevalence. In the full model (model 4), the results show that although the effect of indoor air pollution on children's ARI was slightly reduced, it remained significant. In this adjusted model, children living in households using biomass fuels were associated with 10% increased the risk of ARI (OR: 1.10, 95% CI: 1.01–1.20). Children living in households without a separate kitchen had 22% higher likelihood of ARI (OR: 1.22, 95% CI: 1.14–1.30). Moreover, smoking behavior in households was associated with 6% increased risk of ARI (OR: 1.06, 95% CI: 1.00–1.12) among under-five children.

**Table 2:** Binary logistic regression analysis assessing the impact of indoor air pollution on ARI of under-five children.

Variables	Model 1	Model 2	Model 3	Model 4
	OR (95% CI)	OR (95% CI)	OR (95% CI)	OR (95% CI)
Type of cooking fuel				
Clean fuel (Ref.)				
Biomass fuel	1.20 (1.13–1.26)**	1.16 (1.09–1.23)**	1.15 (1.07–1.22)**	1.10 (1.01–1.20)*
Separate kitchen				
Yes (Ref.)				
No		1.22 (1.15–1.29)**	1.21 (1.14–1.28)**	1.22 (1.14–1.30)**
Smoking behavior				
No (Ref.)				
Yes			1.08 (1.02–1.15)**	1.06 (1.00–1.12)*
Age of children (months)				
0–11 (Ref.)				
12–35				0.91 (0.84–0.98)**
36–59				0.68 (0.63–0.74)**
Sex of children				
Male (Ref.)				
Female				0.85 (0.80–0.90)**
Birth order				
<3 (Ref.)				
≥3				1.19 (1.10–1.28)**
Maternal age (years)				
15–24 (Ref.)				
25–34				0.89 (0.83–0.96)**
35–49				0.87 (0.77–0.99)*
Maternal education				
No education (Ref.)				
Primary				1.32 (1.19–1.45)**
Secondary				1.19 (1.09–1.28)**
Higher				1.21 (1.06–1.38)**
Caste				
SC/ST (Ref.)				
OBC				0.98 (0.91–1.06)
Other				1.04 (0.96–1.14)
Religion				
Hindu (Ref.)				
Muslim				1.16 (1.07–1.26)**
Other				0.96 (0.83–1.11)
Wealth quintile				
Poorest (Ref.)				
Poorer				0.93 (0.85–1.02)
Middle				0.89 (0.81–0.99)*
Richer				0.92 (0.83–1.04)
Richest				0.91 (0.79–1.06)
Any HH member suffers from TB				
No (Ref.)				
Yes				1.12 (0.90–1.40)
Household crowding				
≤3 persons per room (Ref.)				
>3 persons per room				1.02 (0.95–1.08)

Ref.: Reference category; OR: Odds ratio; CI: Confidence interval.

* p<0.05.

** p<0.01.

A number of socio-demographic factors were found to be significantly associated with children's ARI prevalence. Children aged 12–35 months (OR: 0.91, 95% CI: 0.84–0.98) and 36–59 months (OR: 0.68, 95% CI: 0.63–0.74) were less likely to suffer from ARI compared to children aged 0–11 months. Female children were less likely (OR: 0.85, 95% CI: 0.80–0.90) than male children to suffer from ARI. Children in birth order of three or above had an increased risk of having ARI (OR: 1.19, 95% CI: 1.10–1.28) compared to those of birth order below three. The likelihood of ARI decreased with the increasing age of mothers. Children of mothers aged 25–34 years (OR: 0.89, 95% CI: 0.83–0.96) and 35–49 years (OR: 0.87, 95% CI: 0.77–0.99) were less likely to have ARI than those whose mothers aged 15–24 years. Surprisingly, maternal education was positively correlated with the ARI prevalence of children. Muslim children had a higher vulnerability of ARI (OR: 1.16, 95% CI: 1.07–1.26) as compared to Hindus. Children were at greater risks of ARI if any household member had history of suffering from TB; however this result was not significant in the adjusted model (Table 2).

Table 3 provides additional analysis for assessing the impact of indoor air pollution on children's ARI prevalence accounting interaction of the type of cooking fuel and the presence of separate kitchen in the household. The results show that children living in households using clean fuels without a separate kitchen were associated with 27% increased risk of ARI (AOR: 1.27, 95% CI: 1.13–1.41). Results also indicate that households using biomass fuels with separate kitchen increased the likelihood of ARI among children by 13% (AOR: 1.13, 95% CI: 1.01–1.26). Furthermore, the use of biomass fuels in the house without a separate kitchen was found to be the most critical risk factor of ARI among children. The combined effects of biomass fuels and no separate kitchen increased the risks of ARI among children by 35% (AOR: 1.35, 95% CI: 1.21–1.51).

**Table 3:** Interaction analysis showing the effects of type of cooking fuel and separate kitchen on children's ARI.

Indoor air pollution	COR (95% CI)	AOR (95% CI)
Cooking fuel × Separate kitchen		
Clean fuel × having separate kitchen (Ref.)		
Clean fuel × no separate kitchen	1.26 (1.14–1.39)**	1.27 (1.13–1.41)**
Biomass fuel × having separate kitchen	1.18 (1.09–1.28)**	1.13 (1.01–1.26)*
Biomass fuel × no separate kitchen	1.42 (1.32–1.52)**	1.35 (1.21–1.51)**

Ref.: Reference category; COR: Crude odds ratio; AOR: Adjusted odds ratio: CI: Confidence interval.

* p<0.05.

** p<0.01.

Adjusted model was controlled for smoking behavior, age of child, sex of child, birth order, maternal age, maternal education, caste, religion, wealth quintile, any household member suffers from TB, and household crowding.

## Discussion

Acute respiratory infections are serious public health problems in India, leading to high neonatal and child mortality. According to the latest NFHS-4 estimates, over two-thirds of households are using biomass fuels for cooking in India [28]. Despite the implementation of early childhood disease control and survival programs, the burden of respiratory illnesses remains widespread in India. Around 3% of children suffered from ARI in the past two weeks preceding the NFHS-4 survey. The present study provides substantial evidence on the impact of indoor air pollution on respiratory illnesses of under-five children.

The findings of this study show that biomass fuels increased the risk of children's ARI prevalence by 10%, independent of the presence of separate kitchen, smoking behavior, and socio-demographic factors. Studies have consistently indicated that the combustion of biomass fuels emits many toxic pollutants, and children who are mostly exposed to these pollutants suffer from adverse health outcomes, including respiratory infections [22, 30, 31]. Our study also reveals that children living in households without a separate kitchen were 22% more likely to suffer from ARI than those having a separate kitchen. Children are more prone to the exposure of particulate matter when there is no separate kitchen for cooking. Studies have indicated that environmental tobacco smoke is an important risk factor for children's ARI [26]. Our present study also shows that smoking of tobacco inside the house increased the susceptibility of ARI among children. Considering the role of separate kitchen in determining ARI prevalence of children, our study conducted an additional analysis by applying the interaction of cooking fuels and the presence of separate kitchen in the household. The findings indicate that the facility of a separate kitchen strongly modified the impact of cooking fuels on children's ARI. The current study findings are in line with several previous studies from developing countries.

An earlier study conducted in India revealed that children living in households using solid fuels were associated with 78% higher risk of life-threatening respiratory illnesses compared to households using cleaner fuels [32]. Similarly, Mishra et al. [26] found that both biomass cooking fuels and environmental tobacco smoke significantly raised the vulnerabilities of ARI among under-five children. In Bangladesh, the use of in-house biomass fuels increased the risk of respiratory symptoms of children by 18%, after adjusting for residential factors, maternal and child characteristics [33]. Children living in households using solid fuels were 1.19 times more likely to suffer from ARI compared to those living in households using cleaner fuels for cooking in Afghanistan [34]. In Zimbabwe, high pollutant fuels (i.e., wood, dung, or straw) increased the likelihood of children's ARI by more than two-folds compared to low pollutant fuels [35]. A cross-sectional study conducted in an urban slum of Ethiopia reported that children living in households using biomass fuels were almost three times more likely to suffer from ARI compared to children living in households using cleaner fuels [24].

Socio-demographic factors also have a significant impact on children's ARI prevalence. The results demonstrate that younger children are more prone to the risk of infections because younger children tend to spend more time with their mothers in the kitchen, where they get exposed to pollutant air [34]. Moreover, the weak immune system and underdeveloped lungs during infant age lead to the high vulnerability of respiratory infections [19]. In consistent with an earlier study [26], we have found that female children are less likely to suffer from ARI compared to male children. It might be because of the mothers often keep boys in the kitchen while cooking that exposing them to air pollutants, which in turn increases the risk of lung infections [26].

However, our present study is not free from limitations. First, we could not assess the cause-effect relationship between indoor air pollution and ARI of children due to the cross-sectional design of the study. Second, this study is based on secondary data, which is self-reported and retrospective. Therefore, the data are subject to recall bias. Third, we have used cooking fuels, the presence of separate kitchen and smoking behavior as a proxy measure for children's exposure to indoor air pollution instead of the actual concentration of particulate matter due to the limitation of data. The association needs to be validated using direct measures of indoor air pollution. However, this study contributes to effective policy interventions addressing the issue of respiratory infections among children in a developing country like India, where the majority of households still rely on vulnerable biomass fuels for cooking.

## Conclusion

The findings of our study indicate that indoor air pollution significantly increased the risks of ARI among under-five children. Moreover, children of socio-economically vulnerable groups mostly suffered from respiratory illnesses. Policymakers should pay attention to reduce the exposure of indoor air pollution, particularly among vulnerable households. The government should come forward to promote cleaner fuels for cooking, such as LPG and electricity, to minimize exposure to indoor pollution. Besides, spreading awareness about the health risks of smoking behavior could help to reduce the burden of ARI among children. Further research is needed using longitudinal data to explore the potential mechanism mediating the association between indoor air pollution and respiratory infections of children.
